# Genome‐wide single‐nucleotide polymorphism data reveal cryptic species within cryptic freshwater snail species—The case of the *Ancylus fluviatilis* species complex

**DOI:** 10.1002/ece3.3706

**Published:** 2017-12-16

**Authors:** Martina Weiss, Hannah Weigand, Alexander M. Weigand, Florian Leese

**Affiliations:** ^1^ Aquatic Ecosystem Research University of Duisburg‐Essen Essen Germany; ^2^ Centre for Water and Environmental Research (ZWU) University of Duisburg‐Essen Essen Germany; ^3^ Musée National d'Histoire Naturelle Luxembourg Luxembourg

**Keywords:** gastropoda, mito‐nuclear discordance, molecular species delimitation, RAD‐seq

## Abstract

DNA barcoding utilizes short standardized DNA sequences to identify species and is increasingly used in biodiversity assessments. The technique has unveiled an unforeseeably high number of morphologically cryptic species. However, if speciation has occurred relatively recently and rapidly, the use of single gene markers, and especially the exclusive use of mitochondrial markers, will presumably fail in delimitating species. Therefore, the true number of biological species might be even higher. One mechanism that can result in rapid speciation is hybridization of different species in combination with polyploidization, that is, allopolyploid speciation. In this study, we analyzed the population genetic structure of the polyploid freshwater snail *Ancylus fluviatilis*, for which allopolyploidization was postulated as a speciation mechanism. DNA barcoding has already revealed four cryptic species within *A. fluviatilis* (i.e., *A. fluviatilis* s. str., *Ancylus* sp. A–C), but early allozyme data even hint at the presence of additional cryptic lineages in Central Europe. We combined COI sequencing with high‐resolution genome‐wide SNP data (ddRAD data) to analyze the genetic structure of *A. fluviatilis* populations in a Central German low mountain range (Sauerland). The ddRAD data results indicate the presence of three cryptic species within *A. fluviatilis* s. str. occurring in sympatry and even syntopy, whereas mitochondrial sequence data only support the existence of one species, with shared haplotypes between species. Our study hence points to the limitations of DNA barcoding when dealing with organismal groups where speciation is assumed to have occurred rapidly, for example, through the process of allopolyploidization. We therefore emphasize that single marker DNA barcoding can underestimate the true species diversity and argue in strong favor of using genome‐wide data for species delimitation in such groups.

## 1 INTRODUCTION

Biodiversity assessments and ecological status monitoring typically depend on reliable species identification. However, few concepts in biology have been subject to such controversial and semantic discussions as the “species” concept (reviewed e.g. in de Queiroz, 1998). While many different concepts exist, according to de Queiroz (2007), basically all contemporary used species concepts agree in defining species as separately evolving metapopulation lineages and they only disagree in secondary criteria, defining different properties acquired by lineages during the cause of divergence. This seems intuitive, given the complex set of, for example, diagnostic behavioral, ecological, genetic, and/or phenotypic differences we can observe among sister lineages, and which may arise at different times in the speciation process and are thus no necessities for defining the species category (de Queiroz, 2007). As speciation is not always correlated with morphological differentiation (Daïnou et al., 2016), morphological species identification can be impeded by low phenotypic differentiation or even so‐called morphological stasis, high intraspecific phenotypic variability with only the phenotypically “extreme” forms being recognizable or an inadequate set of potentially diagnostic characteristics (Bickford et al., 2007; Fontaneto, Giordani, Melone, & Serra, 2007; Weigand et al., 2013, 2017). To deal with this problem, molecular markers are increasingly integrated in the process of species identification, an approach termed “DNA barcoding”. In animals, the standard barcoding marker comprises a fragment of the mitochondrial cytochrome c oxidase subunit I (COI) gene (Hebert, Cywinska, Ball, & deWaard, 2003). DNA barcoding has led to the detection of a great number of previously overlooked morphologically cryptic species (e.g., Johnson, Warén, & Vrijenhoek, 2008; Kane, Stothard, Emery, & Rollinson, 2008; Katouzian et al., 2016; Nakano & Spencer, 2007; Weiss, Macher, Seefeldt, & Leese, 2014). Besides, studies focusing on the ecology of cryptic species revealed significant differences in their ecological demands and robustness against stressors (e.g., Feckler, Thielsch, Schwenk, Schulz, & Bundschuh, 2012; Macher et al., 2016), emphasizing the importance of correct species identification. However, if speciation has occurred relatively recently and rapidly or is still ongoing, recognizing and defining species boundaries becomes difficult (Altermann, Leavitt, Goward, Nelsen, & Lumbsch, 2014; Shaffer & Thomson, 2007). Focusing on single species concepts (or single secondary criteria sensu de Queiroz, 2007) will hence neglect the complex nature of speciation, because the different diagnostic criteria might or might not have been acquired yet (de Lafontaine, Prunier, Gérardi, & Bousquet, 2015). This highlights the importance of differentiating between primary and secondary properties for defining species (de Queiroz, 2007). When dealing with recent or ongoing speciation, also COI will likely fail in detecting species (Meyer & Paulay, 2005; Moritz & Cicero, 2004), as will slower evolving nuclear genes, because time since speciation was too short to accumulate fixed and diagnostic interspecific differences. Here, the use of genome‐wide single‐nucleotide polymorphism (SNPs) data can be a solution (Daïnou et al., 2016; Razkin et al., 2016; Shaffer & Thomson, 2007). One powerful method to generate a set of informative genome‐wide SNPs applicable for nonmodel organisms is double‐digest restriction site‐associated DNA (ddRAD) sequencing (Peterson et al., 2012). Multilocus sequencing data as, for example, obtained by ddRAD sequencing can provide unprecedented and accurate insights into species delimitation and the process of speciation (e.g., Altermann et al., 2014; Card et al., 2016; Knowles & Carstens, 2007; Weisrock et al., 2010; Yang & Rannala, 2010). These methods can therefore be highly useful when dealing with taxa, where taxonomy is complicated like in the form group *Ancylus fluviatilis* (O. F. Müller, 1774), which has undergone a complex taxonomic history. Whereas Hubendick (1970) in his comprehensive revision only recognized a single and widespread *A. fluviatilis* in Europe creating a multitude of synonymies, the integration of molecular data by Pfenninger, Staubach, Albrecht, Streit, and Schwenk (2003) and Albrecht, Trajanovski, Kuhn, Streit, and Wilke (2006) revealed a total of four cryptic species for this morphospecies. The entities of this cryptic species complex were henceforth treated as *A. fluviatilis* sensu stricto (or Clade 1 in Pfenninger et al. (2003) with specimens collected from the type locality in Ilm, Germany) as well as Clade 2–4 and Clade A–C, respectively. Interestingly, early allozyme studies have found three distinct and reproductively more or less isolated nuclear strains within *A. fluviatilis* in Central Germany (Städler, 1997; Streit et al., 1994). Those were not detected by DNA barcoding as, according to our present knowledge, only *A. fluviatilis* s. str. (or Clade 1) occurs North of the Alps, whereas the other taxa display wider Mediterranean distributions (Clade B and C) or are endemic to south Portugal (Clade A). Yet, the finding of three potentially reproductively isolated strains in Central Germany was not further considered in the updated taxonomy of this species complex.

In this study, we analyzed the population genetic structure of *A. fluviatilis* sensu stricto in a Central German low mountain range (Sauerland, North Rhine‐Westphalia) using COI sequences as well as genome‐wide SNP data obtained by ddRAD sequencing. We here report the presence of further cryptic species within one of the former cryptic species of the *A. fluviatilis* form group (i.e., *A. fluviatilis* sensu stricto, or Clade 1), which are clearly differentiated in nuclear SNP data, but not in mitochondrial sequences. We further discuss potential evolutionary scenarios and general implications for species assignments using mitochondrial gene markers in cases of rapid or ongoing speciation.

## 2 MATERIALS AND METHODS

### 2.1 Sampling and genotyping

Specimens of *A. fluviatilis* were collected in 2013 and 2014 at 14 sampling sites in ten different headwater streams in the Sauerland region (Ruhr catchment) in Central Germany (Table 1). At each sampling site, we collected five specimens at three to five different locations with 200 m distance in between, resulting in 15 to 25 specimens per site and a total sampling size of 275 specimens.

**Table 1:** Sampling sites and number of specimens used in the analyses (*n*)

Site	Stream name	Year	Latitude (WGS84)	Longitude (WGS84)	*n*
VR12	Ennepe	2013	51.170817	7.495388	25
VR11	Refflingser Bach	2013	51.410751	7.654124	20
QB11	Oester	2013	51.158813	7.752986	20
VR20	Oester	2014	51.155842	7.743871	20
QB27	Schürenbach	2014	51.333002	8.226863	25
QB24	Hengsbecker Bach	2014	51.232291	8.173064	20
QB17	Ilpe	2013	51.235900	8.220648	15
QB23	Ilpe	2014	51.229764	8.247229	15
QB22	Kleine Henne	2014	51.325978	8.327106	25
QB20	Elpe	2014	51.343648	8.424339	20
QB12	Elpe	2013	51.269846	8.446041	15
VR17	Palme	2014	51.240981	8.394280	20
VR6	Palme	2013	51.223222	8.400058	20
VR9	Glenne	2013	51.456734	8.434840	15

DNA was extracted from muscle tissue using a salt extraction protocol (Weiss & Leese, 2016). Amplification, purification, and sequencing of the mitochondrial barcoding gene COI were conducted as described in Weiss and Leese (2016), with slight changes in the PCR protocol: Denaturation time for each cycle was extended to 30 s and annealing temperature reduced to 46°C. Bidirectional sequencing was performed on an ABI 3730 sequencer by GATC Biotech (Constance, Germany).

Five ddRAD libraries were generated for the 275 specimens. To avoid laboratory biases depending on batch membership during preparation or sequencing lane, samples belonging to the same sampling site were randomly distributed over batches and lanes. Library preparation was conducted according to the protocol described in Vendrami et al. (2017) with some modifications: The FastDigest restriction enzymes *Csp6I* (GTAC) and *PstI* (CTGCAG; both Thermo Fisher Scientific) were used for the double digestions and the amount of DNA varied between 350 ng and 800 ng, depending on the concentration of the sample after RNA digestion. Furthermore, P7 adapters were modified to fit the overhang generated by *Csp6I* and had no in‐line barcode additional to the index. The expected cut frequency of the restriction enzymes, needed to calculate the amount of adapters during ligation, was estimated on basis of the genome of the freshwater snail *Biomphalaria glabrata* (NCBI accession number: APKA00000000.1.). The *in silico* estimation was conducted using the script genomecut.pl (Rozenberg, https://github.com/evoeco/radtools/) and resulted in an average cut frequency of 306 bp for *Csp6I* and of 9785 bp for *PstI*. In most cases, the PCR was successful when using 2 μl of DNA. If the DNA concentration of a sample after PCR was too low, the PCR was repeated with 1 to 5 μl of DNA template. The total DNA concentration and the concentration of fragments within a range of 308 to 462 bp were measured for each sample on a Fragment Analyzer with the High Sensitivity NGS Fragment Analysis Kit (both Advanced Analytical). The required amount of DNA of each sample for equimolar pooling was calculated on basis of the selected fragment size range as the following final size selection for each library was conducted for this range. The final libraries were sent for sequencing to GATC Biotech AG (Constance, Germany) and were sequenced on an Illumina HiSeq 2500 sequencer using 125 bp paired‐end reads. Details on the ddRAD library preparation of each sample are given in Table S1.

### 2.2 COI data analysis

The obtained sequences of *A. fluviatilis* were assembled and edited in Geneious 8.1.2 (http://www.geneious.com, Kearse et al., 2012) and aligned with MAFFT v7.017 (Katoh & Standley, 2013) as implemented in Geneious using the automatic algorithm selection and default settings. The alignment was trimmed to the shortest sequence used, and haplotype frequencies were determined for the different sampling sites. To link our specimens to the known mitochondrial clades, sequences were blasted against the NCBI database. A median‐joining network (Bandelt, Forster, & Röhl, 1999) was created in PopART v.1.7 (popart.otago.ac.nz) to visualize distances between haplotypes.

### 2.3 ddRAD data analysis

Quality control and trimming of raw reads were performed with Trim Galore! (http://www.bioinformatics.babraham.ac.uk/projects/trim_galore), using a quality value of 15 and a minimum sequence length of 120 bp. The subsequent demultiplexing, trimming of adapter sequences, removing of PCR duplicates, and trimming to similar length (P5 read: 112 bp, P7 read: 106 bp) were conducted using the script preprocess_ddradtags.pl (Schweyen, Rozenberg, & Leese, 2014). Following this, denovo_map.pl of Stacks v. 1.34 (Catchen, Hohenlohe, Bassham, Amores, & Cresko, 2013) was used to identify and genotype loci in the specimens. As increasing the stringency may help to eliminate combining paralogues during SNP discovery analysis when dealing with polyploid genomes (Dufresne, Stift, Vergilino, & Mable, 2014), three different combinations of settings for building loci were used: b1: m3 M3 N5 n3; b2: m3 M2 N4 n2; b3: m3 M4 N6 n4. The parameter *m* defines the number of identical reads needed to build a stack, and *M* defines the maximum distance allowed between stacks within individuals. Changing N alters the maximum number of mismatches for aligning secondary reads to primary stacks, and *n* specifies the number of mismatches between catalog loci. After exporting data with export_sql.pl from Stacks specifying a minimum stack depth of 8, further analyses were conducted using the workflow management tool Snakemake (Köster & Rahmann, 2012), in which different self‐written scripts for data reformatting, filtering, and population genetic analyses were combined. Through the possibility to use wildcards in the Snakemake workflow, datasets resulting from the different Stacks batches and different filter settings could be easily analyzed simultaneously. The Snakemake workflow included the script stacks2fasta.pl (Macher et al., 2015). Further used scripts and the Snakemake workflow are available in BitBucket (https://bitbucket.org/GeneStream_PhD/ddrad_workflow_af). Within the workflow, variable loci were filtered to have a minor allele frequency of 5%, to be present in 95% of specimens and to have 1 to 5 SNPs, of which only one was used for further analyses. After a first analysis (unfiltered datasets), specimens with more than 15% missing data were excluded from downstream analyses (filtered datasets). Basic population genetic statistics like heterozygosity, gene diversity, fixation index (*F*_ST_), and inbreeding coefficient (*F*_IS_) after Weir and Cockerham (1984) were calculated for the unfiltered and filtered datasets for all Stacks settings with the R‐package hierfstat in R v. 3.3.2 (R Core Team, 2015). To analyze the genetic structure in the different datasets, principal component analyses (PCAs, Patterson, Price, & Reich, 2006) were conducted and individual ancestry coefficients were estimated based on sparse non‐negative matrix factorization algorithms (Frichot, Mathieu, Trouillon, Bouchard, & François, 2014), both with the R‐package LEA (Frichot & François, 2015). For the sNMF analyses, 1 to 17 clusters, 30 replicates, and 100,000 iterations per replicate were used. Ploidy was set to four, as *A. fluviatilis* is assumed to be at least tetraploid (Pfenninger et al., 2003). To select the best replicate and the most probable number of clusters (*K*) per dataset, cross‐entropy values were compared between replicates or between clusters, respectively. Additionally, basic population genetic statistics were separately calculated for the single clusters, excluding hybrids and using the same filter settings as for the total dataset.

Further, Neighbor‐joining trees (Saitou & Nei, 1987) were calculated using SplitsTree v. 4.12.3 (Huson & Bryant, 2006) for the three different Stacks batches. The handling of ambiguous states was set to “MatchStates”. To visualize ddRAD cluster as well as COI haplotypes, a map was constructed in QGIS 2.8 (QGIS Development Team, 2015). The stream network layer was provided by the federal state authority LANUV (Gewässerstationierungskarte des Landes Nordrhein‐Westfalen © LANUV NRW (2013)).

## 3 RESULTS

### 3.1 COI pattern

**Figure 1:**
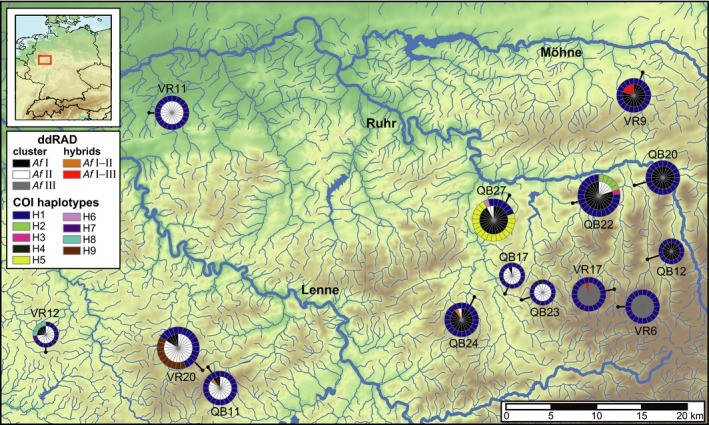
Map of Sauerland region showing COI haplotype composition (outer circle) and ddRAD cluster assignment (inner circle) for individuals of *Ancylus fluviatilis* at the different sampling sites. The size of pie charts is scaled according to the number of analyzed specimens. The red box in the small map in the upper right corner indicates the location of the study area

**Figure 2:**
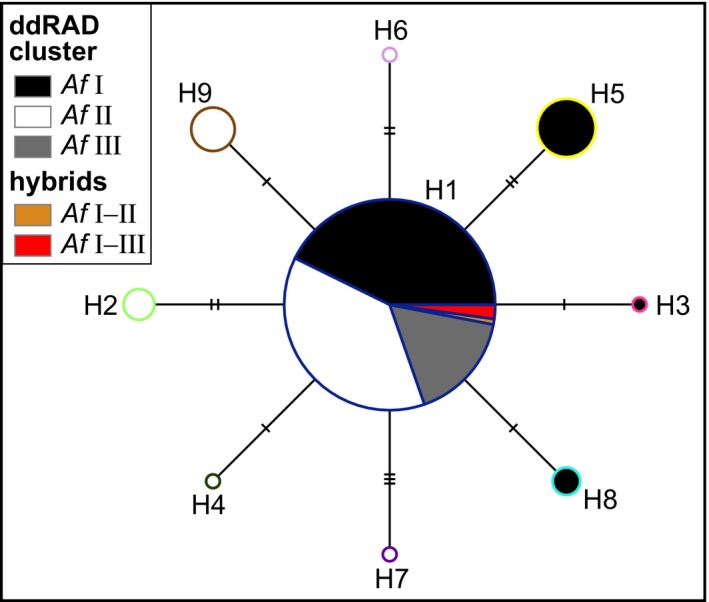
Median‐joining network of COI sequences. The size of circles is scaled according to number of specimens showing the respective haplotype. Small dashes on connecting branches indicate number of differences between haplotypes. Haplotypes are colored according to ddRAD clusters and frame color corresponds with color of haplotypes in Figure 1

The alignment of the 275 sequences had a length of 583 bp containing 13 variable sites, of which four were nonsynonymous substitutions. BLAST searches revealed that all individuals sequenced belonged to the mitochondrial Clade 1, representing *A. fluviatilis* sensu stricto (Albrecht et al., 2006; Pfenninger et al., 2003). In total, nine haplotypes were detected. The most common haplotype (H1) was found at all sampling sites and in 85.1% of all specimens. At nine sampling sites, H1 was found exclusively, the other five sites had additional private haplotypes with varying frequencies, and thus, no geographic pattern was detected in the COI dataset (Figure 1). All haplotypes clustered around the main haplotype in a star‐like pattern, and the maximum distance between haplotypes was five mutations (Figure 2). Haplotypes for each sample and accession numbers of haplotypes are given in Table S2.

### 3.2 ddRAD pattern

Using all 275 specimens in the analysis resulted in 875 to 2319 loci depending on the Stacks settings (Table 2). The number of loci increased when allowing more mismatches for primary and secondary reads. Excluding individuals with more than 15% missing data (8 for b1 and b3, 7 for b2) resulted in a similar overall pattern with the number of loci varying between 1,070 and 2,838. In all datasets, the observed heterozygosity was high with values ranging from 0.42 to 0.58, resulting in negative *F*_IS_ values between −0.69 and −0.53.

**Table 2:** Population genetic statistics and PCA results of ddRAD data for the different Stacks settings (b2: m3 M2 N4 n2, b1: m3 M3 N5 n3, and b3: m3 M4 N6 n4). Ho, Hs, and Ht are observed heterozygosity, within‐population gene diversity, and overall gene diversity, respectively. *F*_ST_ and *F*_IS_ were calculated according to Weir and Cockerham (1984)

Dataset	Stacks setting	*n*	# Loci	Ho	Hs	Ht	*F*_ST_	*F*_IS_	Sig. PCA axes	% Variance explained by		
										1. axis	2. axis	Other axes
Unfiltered	b2	275	875	0.45	0.28	0.40	0.30	−0.57	27	39.9	8.4	<2.0
Unfiltered	b1	275	1,753	0.54	0.32	0.42	0.24	−0.65	31	36.5	6.2	<1.6
Unfiltered	b3	275	2,319	0.58	0.34	0.43	0.20	−0.69	34	33.2	5.6	<1.7
Filtered	b2	268	1,070	0.42	0.27	0.39	0.32	−0.53	26	40.7	9.3	<2.1
Filtered	b1	267	2,135	0.50	0.31	0.42	0.27	−0.62	30	38.9	6.8	<1.6
Filtered	b3	267	2,838	0.55	0.33	0.43	0.23	−0.66	28	35.7	6.0	<1.6

**Figure 3:**
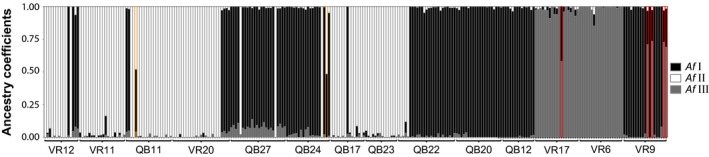
Graphical illustration of ancestry estimates for filtered ddRAD dataset for b2 Stacks settings and *K* = 3 (cross‐entropy = 0.23). Estimated ancestry coefficients for each individual are represented by vertical bars, and population association is indicated by curly brackets below the plot. *Af* Ι–ΙΙ hybrids are highlighted with orange frames, *Af* Ι–ΙΙΙ hybrids with red frames. Cluster are colored according to species *Af* Ι, *Af* ΙΙ, and *Af* ΙΙΙ

**Figure 4:**
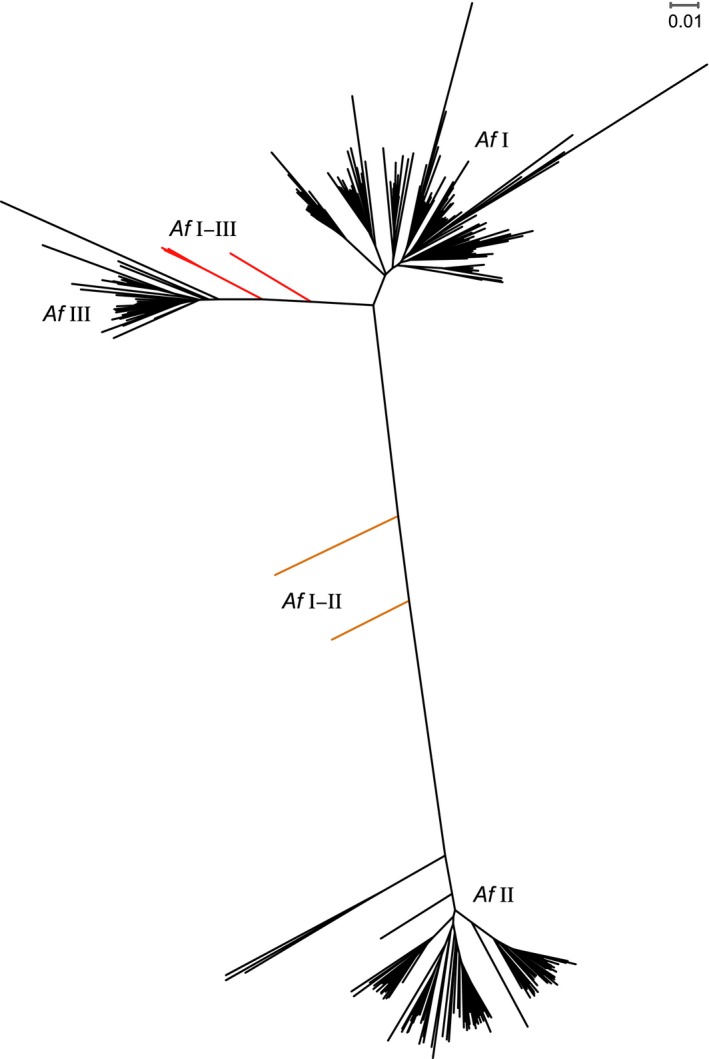
Neighbor‐joining tree for the filtered ddRAD dataset for b2 Stacks settings. *Af* Ι–ΙΙ hybrids are colored in orange and *Af* Ι–ΙΙΙ hybrids in red

To get a first overview of the population structure, a principle component analysis (PCA) was conducted. In all datasets, the PCA indicated 26 to 34 significant PCA axes, from which the respective first two axes explained most of the variance (Table 2). The first axis explained 33.2% to 40.7% of the variance and the second axis 5.6% to 9.3%, respectively. This resulted in a clear clustering of specimens for all datasets, in which a major proportion of the variance was explained by these axes (Figure S1). An sNMF analysis was conducted to analyze the population structure in more detail. According to the cross‐entropy criterion, *K* = 3 was the best number of clusters in all datasets, because cross‐entropy did not decrease much for values greater than 3 (Figure S2). Nevertheless, the low values for higher cluster numbers indicate further population structure within the three main clusters, which were consistent with the PCA clusters. Specimens with membership probabilities higher than 80% for one of the clusters were assigned to the respective cluster, whereas specimens not matching this threshold were treated as hybrids. The individual membership probabilities between datasets differed slightly, but the individual assignment to one of the three clusters, or as a hybrid between two of the clusters, was consistent for all specimens. When analyzing the unfiltered dataset, specimens excluded in the filtered dataset due to high proportions of missing data could still be assigned to one of the clusters with high probability. The assignment of specimens to the three clusters for the filtered dataset b2 is shown in Figure 3, and clusters are further named *A. fluviatilis* Ι, ΙΙ, and ΙΙΙ, respectively, or *Af* Ι, *Af* ΙΙ, and *Af* ΙΙΙ for better readability. Of the 275 specimens, 123 were assigned to *Af* Ι, 106 to *Af* ΙΙ, and 39 to *Af* ΙΙΙ. Further few hybrids were detected: two between *Af* Ι and *Af* ΙΙ and five between *Af* Ι and *Af* ΙΙΙ. Whereas the *Af* Ι–ΙΙΙ hybrids of the population VR9 clustered approximately to 75% to *Af* ΙΙΙ, all other hybrids clustered more or less to 50% to the respective cluster. The three clusters and seven hybrids are also visible in the neighbor‐joining tree (Figure 4), with *Af* Ι and *Af* ΙΙΙ being closer related than both to *Af* ΙΙ. Between the three Stacks settings, some differences were visible within clusters, but the association to the respective cluster was consistent between settings and also with the sNMF analysis; therefore, only the tree for b2 Stacks settings is shown.

*Af* Ι was found at ten sampling sites, *Af* ΙΙ at nine sites, and *Af* ΙΙΙ at two sites (Figure 1). At seven of the 14 sampling sites, *Af* Ι and *Af* ΙΙ were found in syntopy and the two *Af* Ι–ΙΙ hybrids were found at two of those sites (QB11 and QB24). *Af* ΙΙΙ did not co‐occur with *Af* Ι or *Af* ΙΙ. However, four of the *Af* Ι–ΙΙΙ hybrids were found at a site where otherwise only *Af* Ι was found (VR9) and the fifth hybrid at one of the *Af* ΙΙΙ‐sites (VR17). Generally, *Af* Ι and *Af* ΙΙΙ were more often found in the eastern part, whereas *Af* ΙΙ was more frequent in the western part of the sampling area, but there was no clear geographic distribution pattern.

Analyzing the different clusters separately resulted in 970 to 2414 loci for *Af* Ι, 1060 to 2705 for *Af* ΙΙ, and 766 to 1886 for *Af* ΙΙΙ depending on the Stacks settings (Table 3). Within all clusters, the observed heterozygosity was very high with values between 0.6 and 0.78 resulting in negative *F*_IS_ values ranging between −0.81 and −0.60.

**Table 3:** Basic population genetic statistics of ddRAD data for the different nuclear cluster and respective Stacks settings (b2: m3 M2 N4 n2, b1: m3 M3 N5 n3, and b3: m3 M4 N6 n4). Ho, Hs, and Ht are observed heterozygosity, within‐population gene diversity, and overall gene diversity, respectively. *F*_ST_ and *F*_IS_ were calculated according to Weir and Cockerham (1984)

Nuclear cluster	Stacks setting	*n*	# Loci	Ho	Hs	Ht	*F*_ST_	*F*_IS_
*Af* Ι	b2	119	970	0.60	0.35	0.39	0.13	−0.70
*Af* Ι	b1	119	1,824	0.70	0.39	0.43	0.09	−0.76
*Af* Ι	b3	119	2,414	0.74	0.41	0.44	0.07	−0.78
*Af* ΙΙ	b2	104	1,060	0.69	0.40	0.44	0.10	−0.75
*Af* ΙΙ	b1	104	2,065	0.76	0.42	0.45	0.07	−0.80
*Af* ΙΙ	b3	104	2,705	0.78	0.43	0.46	0.06	−0.81
*Af* ΙΙΙ	b2	37	766	0.66	0.41	0.42	0.04	−0.60
*Af* ΙΙΙ	b1	37	1,408	0.75	0.44	0.44	0.02	−0.70
*Af* ΙΙΙ	b3	37	1,886	0.77	0.45	0.45	0.02	−0.72

### 3.3 Comparison of the two marker systems

Comparing the COI dataset and the nuclear dataset revealed no correspondence between nuclear and mitochondrial differentiation, as the main haplotype H1 was found in specimens of all three nuclear clusters and differentiation in the COI sequences was generally low (Figure 2). Additional to the main haplotype, we detected three private haplotypes for *Af* Ι and five private haplotypes for *Af* ΙΙ. For *Af* ΙΙΙ, only the main haplotype was found, which applies also for all hybrids. The haplotypes, which were private for the different nuclear cluster, were also only found at one sampling site.

## 4 DISCUSSION

Using genome‐wide SNP data, we revealed an unexpectedly high differentiation within the freshwater snail *A. fluviatilis* sensu stricto, which is one species of a cryptic species complex delimited by molecular taxonomy (Albrecht et al., 2006; Pfenninger et al., 2003). The strong differentiation of three clusters was already visible in the PCA. Comparably high proportions of variance between groups had been found in other taxa for inter‐ and not intraspecific comparisons (e.g., Christe et al., 2016; Stemshorn, Reed, Nolte, & Tautz, 2011; Weigand et al., 2017). The strong clustering was further confirmed with the sNMF approach (Frichot et al., 2014), where individual ancestry coefficients are calculated comparable to methods such as STRUCTURE (Pritchard, Stephens, & Donnelly, 2000). This approach has been recommended as a useful approach for delimiting species in recent radiations (Shaffer & Thomson, 2007). In the ancestry coefficient plots, it became apparent that clustering was not overall congruent with populations sampled at different sites, but that at half of the sites two different clusters were found in syntopy. Finally, the Neighbor‐joining tree revealed that the three genetic clusters were distinct and strongly differentiated lineages with *Af* Ι and *Af* ΙΙΙ closer related to each other than both with *Af* ΙΙ. A few hybrids were visible between lineages, but no further admixture present. This indicates that the nuclear clusters represent independently evolving lineages, with lineages defined as ancestor–descendent series (Hull, 1980; Simpson, 1961). Therefore, following the unified species concept of de Queiroz (2007), where species are defined as separately evolving metapopulation lineages, the three distinct lineages we found within *A. fluviatilis* s. str. very likely represent different species. Reproductive isolation is not assumed as a necessary species delimiting criterion, and therefore, the rare hybrids we detected do not contradict the assumptions of genetic clusters being evolutionary distinct species, further elucidated in, for example, Harrison and Larson (2014) and Mallet (2007). In addition to the strong differentiation, the syntopic occurrence of the different clusters in combination with very low rates of gene flow can be seen as further support for defining the genetic clusters as separate species (Daïnou et al., 2016). Our findings are congruent with early molecular studies conducted in another region in Central Germany using allozymes (Städler, 1997) and RAPDs (Kuhn & Schierwater, 1993). The three lineages observed in the former studies were described as different strains within *A. fluviatilis* sensu Hubendick (1970), even though it was hypothesized that they were products of separate ancient hybridizations and polyploidizations among genetically differentiated progenitors (Städler, Loew, & Streit, 1996; Städler, 1997; Streit et al., 1994), indicating that they evolved separately. As we did not analyze the same populations and used another marker system, we cannot directly link our results to these studies. However, it is very likely that the three strains detected previously within *A. fluviatilis* are those we report here from the Ruhr area, yet a direct comparison is needed for future validation.

In contrast to the genome‐wide SNP data, mitochondrial COI sequence data only suggested the presence of one species in the study area. One reason for the lack of COI differentiation could be that time since speciation onset was too short for divergence to already be apparent in the COI gene. Moritz and Cicero (2004) predicted that new or rapidly diverged species will be overlooked when focusing on mtDNA divergence for species recognition. One mechanism that can result in rapid and even instantaneous speciation is hybridization of different species combined with polyploidization (i.e., allopolyploid speciation), because the originating new hybrid species are often reproductively isolated from their progenitors and can have advantages over their parents such as heterozygote advantage or extreme phenotypic traits (e.g. Abbott et al., 2013; Mallet, 2007). This speciation mechanism has been hypothesized for different hermaphrodite snails including *A. fluviatilis* (e.g., Goldman, LoVerde, & Chrisman, 1983; Streit et al., 1994). The species complex of *A. fluviatilis* is generally considered to be polyploid with known ploidy levels of tetraploid (Patterson & Burch, 1978), hexaploid (Baršiene, Tapia, & Barsyte, 1996), and octoploid cytotypes (Burch, 1962). However, it is not known if chromosome numbers differ between or within species. In our study, we did not analyze the ploidy level of specimens, but the data provide indirect evidence that all three species are polyploid. In allopolyploid organisms, each chromosome is represented by at least two sets of divergent chromosomes, where chromosomes originating from the different ancestral species are called homoeologues (Dufresne et al., 2014). If the two ancestral genomes are similar enough, homoeologous loci will be clustered together as one locus in the Stacks analysis and the two fixed actually homozygous locus pairs inherited from the different progenitors will appear heterozygous in the analysis. This “fixed heterozygosity” can then lead to high observed heterozygosity increasing with more relaxed Stacks settings. The fact that we found such high values even with strict clustering settings indicates that homoeologous loci could not be fully disentangled by the analysis. This on the other hand implies that the ancestral species were probably closely related and/or that the hybridization has happened relatively recently as otherwise homoeologous loci should be better separated by the analysis. Inferring population structure of polyploid species can be difficult, because clustering methods such as STRUCTURE (Pritchard et al., 2000) rely on population genetic assumptions like Hardy–Weinberg equilibrium. This is also problematic when dealing with asexual reproduction and inbreeding, which are known to be important in *A. fluviatilis* (Stadler, Loew, & Streit, 1993; Städler, Weisner, & Streit, 1995). However, the methods we used to infer population structure, that is, PCA and sNMF, do not rely on these assumptions and are therefore suitable for the analysis of polyploid data, mixed‐ploidy data, and also for species with high levels of inbreeding (Dufresne et al., 2014; Frichot et al., 2014). In addition, results were consistent across all methods (PCA, sNMF, and Neighbor‐Joining tree) and Stacks settings, implying high reliability of results.

As described above, allopolyploidization can lead to rapid speciation which could already have caused the observed mito‐nuclear discordance. Beside or in combination with this, also the process of allopolyploid speciation itself could have generated the discordance pattern. During allopolyploid speciation, the new polyploid species only inherits the mitochondrial DNA from one ancestor (Evans, Kelley, Tinsley, Melnick, & Cannatella, 2004), which could lead to haplotype sharing between the new species, in particular over short temporal periods. As allopolyploid species mostly originate from multiple hybridization events (e.g., Mable, 2004), sex‐biased hybridization, as was found among two hermaphrodite freshwater snail species of the genus *Physa* (Wethington, Kirkland, & Dillon, 2012), would probably be needed to create the pattern of uniformity in COI sequences we found. Another explanation for the haplotype sharing between species could be that the hybridizing species were not yet differentiated in their mitochondrial genome. This hypothesis is supported by the ddRAD results, which indicated that the progenitor species were probably closely related. However, to disentangle the complicated phylogenetic history and the involved speciation mechanisms of the *A. fluviatilis* species complex, a geographically and taxonomically much broader sampling is needed together with chromosome number estimates for the different species.

In conclusion, we demonstrated that even at a small geographic scale, further overlooked cryptic species can exist within an already recognized cryptic species complex. When relying on the classical mitochondrial COI barcoding approach only, these species may go unnoticed, highlighting that mtDNA divergence is not always sufficient as a criterion for delineating species (Moritz & Cicero, 2004). Our findings are in accordance with the results of Spooner (2009) who found DNA barcoding inappropriate to investigate species boundaries in a taxonomically complicated plant group, where similar speciation mechanisms have been assumed as for *Ancylus*. Therefore, especially in species with great potential for morphologically cryptic species and where polyploidization is assumed to be an important speciation mechanism during evolutionary history, it is essential to validate COI barcoding results with genome‐wide data.

## CONFLICT OF INTEREST

None declared.

## AUTHOR CONTRIBUTIONS

MW, HW, AMW, and FL conceived and designed the study. MW and HW collected the samples and wrote the scripts for ddRAD data analyses. MW performed the laboratory work and data analysis. MW interpreted the data together with HW, AMW, and FL. MW wrote the manuscript, and all authors contributed to the final version of the manuscript.

## DATA ACCESSIBILITY

ddRAD data generated during this study are available at NCBI BioProject with accession number PRJNA389679. COI haplotypes are available in BOLD with accession numbers AFCS001‐17–AFCS009‐17.
