# Supplementary material for: Genome‐wide single‐nucleotide polymorphism data reveal cryptic species within cryptic freshwater snail species—The case of the Ancylus fluviatilis species complex
Source: Ecol Evol. 2017 Dec 16;8(2):1063–72. doi: 10.1002/ece3.3706 (PMC5773296; doi:10.1002/ece3.3706)
Supplement: Supplementary file 1 [file ECE3-8-1063-s001.pdf]

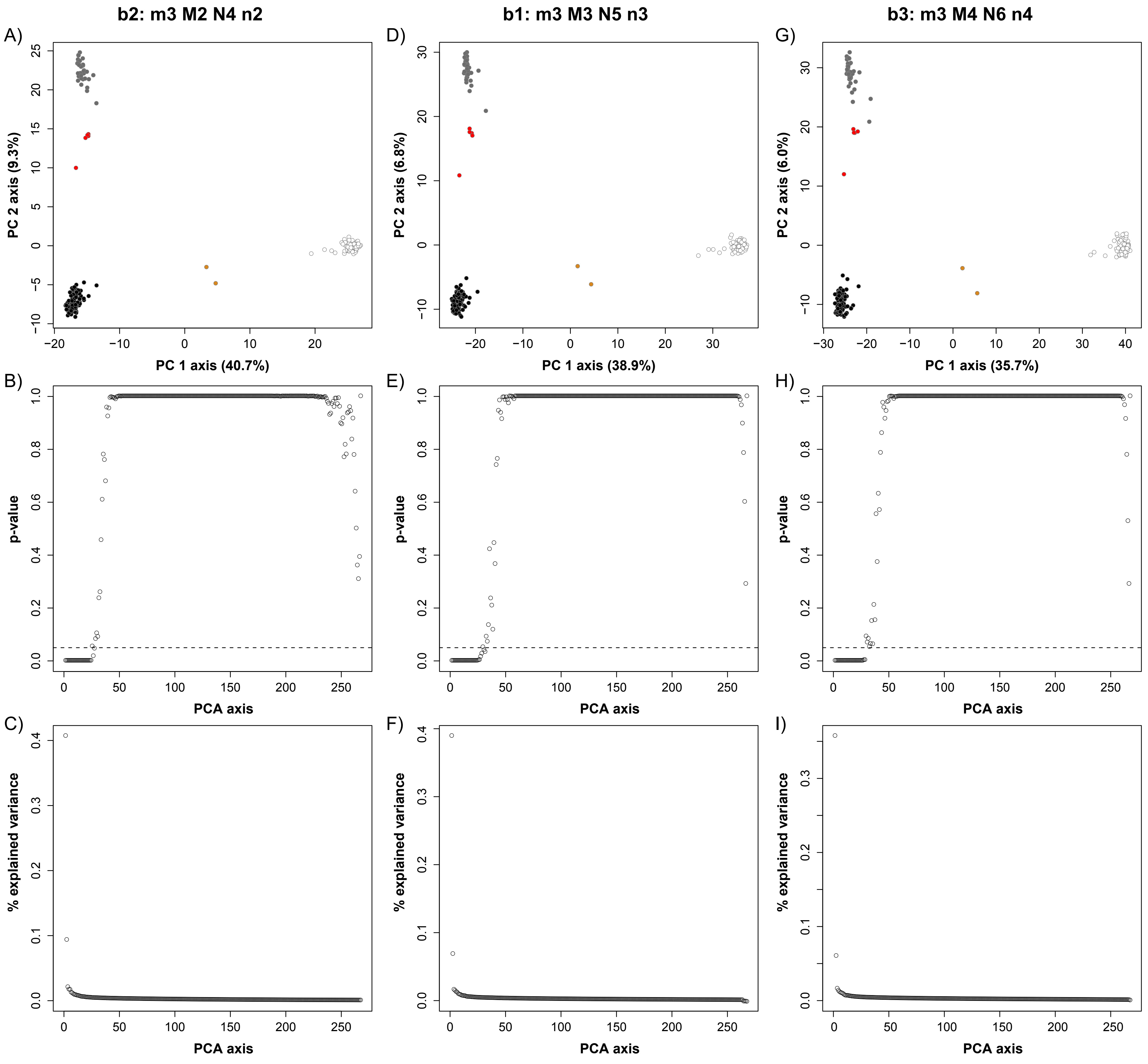

Figure S1. Principle component analysis of the filtered ddRAD dataset. A-C, D-E and G-I show results for the different stacks settings, b2, b1 and b3, respectively. A, D and G are scatterplots showing the first two principal components (PCs), percentage of explained variance by the respective axes is given in brackets. Specimens of *Af* I are colored in black, of *Af* II in white, of *Af* III in grey, *Af* I–II hybrids in orange and *Af* I–III hybrids in red. In B, E and H p-values for all axes are shown with points below the vertical dashed line indicating significance of axes ($p < 0.05$). Plots C, F and I show the percentage of variance explained by each axis.
