# Supplementary material for: Genome‐wide single‐nucleotide polymorphism data reveal cryptic species within cryptic freshwater snail species—The case of the Ancylus fluviatilis species complex
Source: Ecol Evol. 2017 Dec 16;8(2):1063–72. doi: 10.1002/ece3.3706 (PMC5773296; doi:10.1002/ece3.3706)
Supplement: Supplementary file 2 [file ECE3-8-1063-s002.pdf]

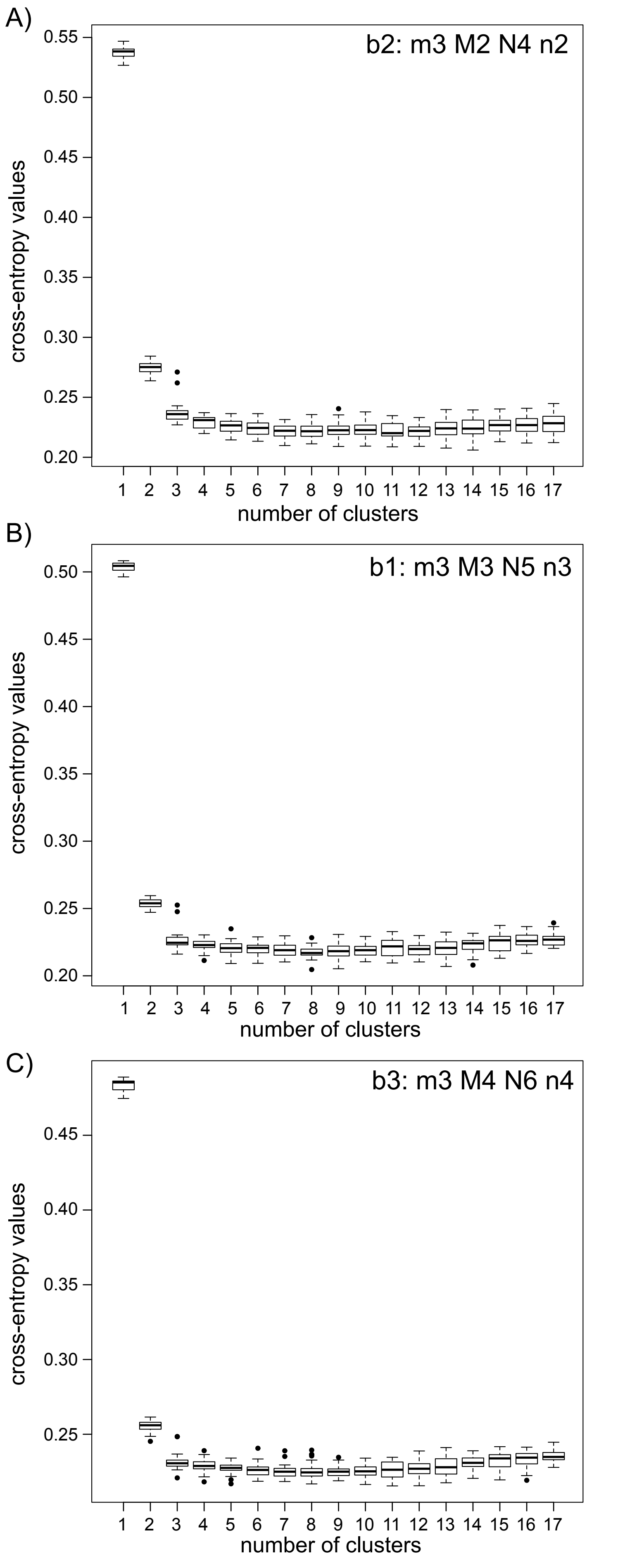

Figure S2. Standard boxplots of cross-entropy values (30 repeats) of sNMF analysis for the filtered ddRAD dataset. A, B and C show boxplots for the different stacks settings.
